# Hydrochemical Characterization of Groundwater Quality for Drinking and Agricultural Purposes: A Case Study in Rafsanjan Plain, Iran

**DOI:** 10.1007/s12403-015-0169-3

**Published:** 2015-06-02

**Authors:** Seyed Javad Hosseinifard, Milad Mirzaei Aminiyan

**Affiliations:** Pistachio Research Institute of Iran, Agricultural Research Education and Extension Organization (AREEO), Rafsanjan, Iran; Graduated Student of Master of Science in Field of Soil Science, Hamedan, Iran

**Keywords:** Groundwater, Irrigation, Water quality, Salinity, Alkalinity, Rafsanjan, Pistachio

## Abstract

One of the important purposes of hydrology is to ensure water supply in accordance with the quality criteria for agricultural, industrial, and drinking water uses. The groundwater is the main source of water supply in arid and semi-arid regions. This study was conducted to evaluate factors regulating groundwater quality in Rafsanjan plain. A total of 1040 groundwater samples randomly were collected from different areas of Rafsanjan. Then, each sample was analyzed for the major ions based on standard methods. The pH, SAR, EC, and TDS parameters and concentrations of Ca^2+^, Mg^2+^, and Na^+^ cations, and Cl^−^, ${\text{CO}}_{3}^{2 - }$, ${\text{HCO}}_{3}^{ - }$ and ${\text{SO}}_{4}^{2 - }$ anions were measured. Also boron concentration in each sample was determined. Although, maximum and minimum values of EC and TDS linked to the Anar-Beyaz area and Eastern Urban, respectively, irrigation water EC condition, however, was critical in the study areas. The pH value in Western Urban was higher than the other areas, and its value for Anar-Beyaz area was lower than the other areas, but pH value is at the optimal level in all the study areas. The results showed that hazard state with respect to Mg was critical except in Koshkoueiyeh and Anar-Beyaz areas, that these areas are marginal for irrigation use with little harm with reference to Mg. From the results, it was concluded that the status of boron concentration in study areas was critical. According to the hydrochemistry diagrams, the main groundwater type in different study areas was NaCl. Groundwater quality was not appropriate for drinking usage, and its status for agricultural practices was unsuitable in these areas.

## Introduction

Water-quality index is one of the most effective tools used in passing information on the quality of water to the concerned citizens and policy makers (Atulegwu and Njoku 2004). Therefore, it is an important parameter for the assessment management of water (Fagbote et al. 2014). The qualities of water bodies vary widely depending on the location and environmental factors. Some of the factors determining the qualities of surface waters and ground waters are the chemical composition of the underlying rocks, soil formations, and the length of time that the water body has been trapped underground (Faniran et al. 2004).

The water quality for irrigation may affect the soils and crops, especially in the saline alkali soil areas. Salinity and sodium hazard indicators can be used as a criterion to find the suitability of irrigation waters (Nishanthiny et al. 2010). The United States Department of Agriculture (USDA) method is the most recognized worldwide, and sodium absorption ratio (SAR) is an effective evaluation index for most of the irrigation waters (Al-Bassam and Al-Rumikhani 2003).

During the last two decades, groundwater quality evaluations in different parts of the world have been studied by various researchers (Çelik and Yildirim 2006; Gallardo and Tase 2007; Partey et al. 2010). Thirumalini and Joseph (2009) have examined various sampling wells to determine regression equations between EC and TDS for fresh water and saltwater in the Thiruvallur district located on the northern border of Tamil Nadu, India. According to their works, a linear correlation exists between these two parameters for fresh water, whereas there is a logarithmic correlation for saltwater. Shah et al. (2008) have compared groundwater quality in Gandhinagar Taluka in India with standard values given by WHO (2011) and have come up with a water-quality index for that area. Arumugam and Elangovan (2009) have studied groundwater quality based on Piper diagram and Na% values for drinking and irrigation purposes in Tirupur region in India. The area under our study (the Rafsanjan plain) has been studied only by a few researchers earlier (Mortazavi et al. 2014).

Groundwater resource assessments and sustainability considerations are of utmost importance in the arid and semi-arid regions, where water is commonly of critical economic and social significance. Continued population growth in Iran is rapidly depleting groundwater supplies in some areas. Intense agricultural and urban development has placed not only a high demand on groundwater resources in arid and semi-arid regions, but also has placed these resources at greater risk of contamination. Groundwater is the primary source of water for domestic, agricultural, and industrial uses in many countries, and its contamination has been recognized as one of the most serious problems in Iran (Jalali 2005, 2007). The groundwater is the main source of water supply in arid and semi-arid regions (Baghvand et al. 2010). Water quality is very important to human health, and the quantity and quality of grains, as it can have effects on soils, crops, and environment (Van der Hoek et al. 2001). Water-quality indices provide a simple and easily understandable tool for managers on the quality and possible uses for irrigation water; however, an individual quality factor alone is not enough to evaluate the irrigation water quality because it could be restrictive, and sometime it could give an unfavorable qualification. The water-quality assessment is mostly based on hydrochemical analysis (Jalali 2007). The hydrochemical study reveals quality of water to determine its suitability for drinking, agricultural, and industrial purposes. Groundwater often consists of seven major chemical elements, e.g., Ca^+2^, Mg^+2^, Cl^−^, ${\text{HCO}}_{3}^{ - }$ Na^+^, K^+^, and ${\text{SO}}_{4}^{2 - }$. The chemical parameters of groundwater play a significant role in classifying and assessing water quality (Jamshidzadeh and Mirbagheri 2011).

Iran is located in a semi-arid area with an average annual precipitation less than one third of that of the world. Furthermore, spatial and temporal distributions of the regional precipitation are not integrated. Iran is one of 27 countries that are likely to face increasing water shortage crises between now and 2025 unless action is taken to reduce current water consumption (Bidhendi et al. 2007). Accordingly, lack of water resources is observed in most parts of the country. Groundwater supplies provide more than half of the total annual water demand in Iran; however, the recharge of such resources is less than half of the total extracted amount (Mortazavi et al. 2014). The uncontrolled groundwater use accompanied by successive famines in recent years has adversely affected the quality and quantity of Iran’s aquifers: particularly in central parts where high-temperature and low-precipitation rates make the conditions more severe (Salehi and Hosseinifard 2012). In the central Iran, during the past few decades, development activities, both in urban and agricultural sectors have rapidly increased; often without adequate planning (Jamshidzadeh and Mirbagheri 2011).

Anthropogenic activities have impacted aquatic ecosystems through alterations in hydrology, introduction of toxic chemicals (including pharmaceuticals and personal care products), increased inputs of nutrients (i.e., cultural eutrophication), and changes to other physicochemical traits of water (e.g., temperature, dissolved/suspended solids) (Paul and Meyer 2001; Asonye et al. 2007). Activities associated with agriculture (Matson et al. 1997) and urbanization (Cole et al. 1993; Caraco 1995) are major contributors to eutrophication and other changes in the chemical composition of aquatic habitats. Understanding the effects of urbanization on aquatic ecosystems has gained increasing importance due to population growth in urban centers and the escalating use of urban aquatic systems for consumptive and nonconsumptive purposes.

The importance of the groundwater resources in the area should not be underestimated because they are the only water resource for drinking and agricultural purposes not only for the people living in this area but also for those who live in the surrounding areas (Baghvand et al. 2010). Despite the lack of alternative water sources, the groundwater hydrogeochemistry of the region remains poorly understood. Salinization and decreasing water levels increase the need for a comprehensive understanding of the groundwater system that would help better management of the resource.

Despite the importance of groundwater in Iran, little is known about the natural phenomena that govern the chemical composition of groundwater or anthropogenic factors that presently affect them (Jalali 2009). The chemical composition of groundwater is controlled by many factors that include composition of precipitation, geological structure, mineralogy of the watersheds and aquifers, and geological processes within the aquifer (Andre et al. 2005). The interaction of all factors leads to various water types. Increased knowledge of geochemical evolution of groundwater in arid and semi-arid regions could lead to improved understanding of hydrochemical systems in such areas, leading to sustainable development of water resources and effective management of groundwater resource.

Our main objective is to study groundwater quality in this area in order to assess the suitability of groundwater for drinking and agricultural uses.

## Materials and Methods

### Study Area

This study was conducted in some wells from different areas of Rafsanjan plain. Rafsanjan plain is located in the northwestern part of Kerman Province, Iran. This area is located between longitude 55°59′30″E and latitude 31°13″N. The mean annual precipitation of this region is less than 100 mm. The mean annual potential evapotranspiration is more than 3000 mm. The six regions including Anar-Beyaz, Koshkoueiyeh, Kabootarkhan, Nooq, Eastern Urban, and Western Urban were selected for the purposes of this study. The area studied occupies about 8000 km^2^, with a mean altitude of 1469 m above mean sea level. Soil moisture and temperature regimes are aridic and thermic, respectively. Groundwater has been used for various purposes, such as drinking, agricultural, domestic, and industrial needs. The most important economic activity of this area is pistachio orchard. In the selected wells, the common irrigation intervals are about 48 days (Hosseinifard et al. 2010).

### Water Sampling and Analysis

**Fig. 1:**
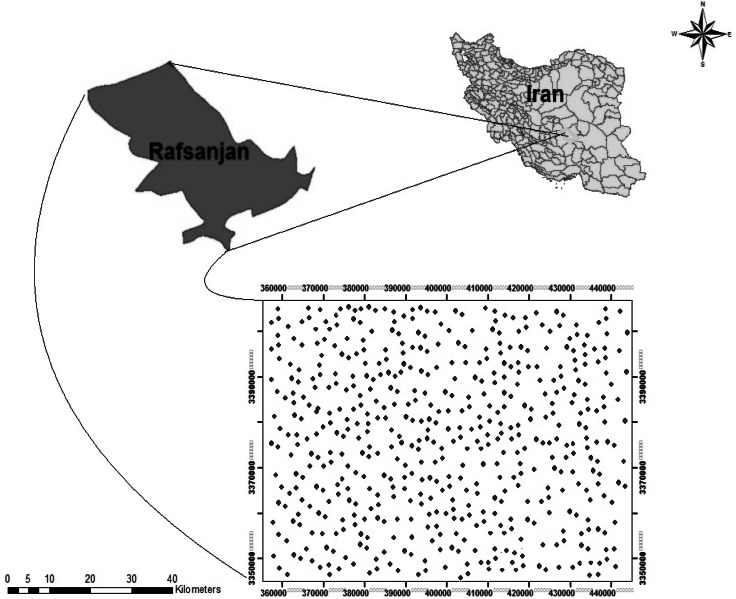
The location of the groundwater sampling wells

In brief, the 1040 groundwater samples randomly were collected in polyethylene bottles (∼250 ml) based on the preliminary field survey from different areas of Rafsanjan plain, during 2012–2014 (Fig. 1). The proportion of each area was 180, 171, 50, 361, 158 and 120 well water samples for Anar-Beyaz, Koshkoueiyeh, Kabootarkhan, Nooq, Eastern area, and Western area, respectively. Then the values of hydrochemical characteristics in each area are reported as mean. Most of these wells supply water for irrigation. Samples were collected after a pumping time of about 30 min from wells. Samples were analyzed in the laboratory for the major ions employing standard methods.

The analyses were carried out after 48 h of collection. All the parameters were determined based on Pansu and Gautheyrou (2006). While the pH and EC values were measured by pH-meter (Metrohm) and an EC-meter (Metrohm), respectively, Calcium (Ca^2+^) and magnesium (Mg^2+^) were determined titrimetrically using standard EDTA. Chloride (Cl^−^) was determined by the standard AgNO_3_ titration method. Carbonate (${\text{CO}}_{3}^{2 - }$) and bicarbonate (${\text{HCO}}_{3}^{ - }$) were determined by titration with HCl. Sodium (Na^+^) was measured by flame photometry, sulfate (${\text{SO}}_{4}^{2 - }$) by spectrophotometric turbidimetry, and boron in water was measured by inductively coupled plasma-atomic emission spectroscopy (ICP-AES). Total dissolved solids (TDS) were computed by multiplying the EC (dS m^−1^) by a factor of 640. The alkalinity (sodium) hazard of water is described by the sodium adsorption ratio (SAR):

1 $$ {\text{SAR}} = \frac{\text{Na}}{{\sqrt {\frac{{\left( {{\text{Ca}} + {\text{Mg}}} \right)}}{2}} }} $$

where all the ionic concentrations are expressed in terms of milli-equivalent per liter (meq l^−1^).

Total hardness (TH) of groundwater was calculated using the following equation (Sawyer et al. 2003):

2 $$ {\text{TH }}\left( {{\text{mg CaCO}}_{ 3} \;{\text{ l}}^{ - 1} } \right) \, = \, \left( {{\text{Ca}}^{ 2+ } + {\text{ Mg}}^{ 2+ } } \right) \, \times { 5}0 $$

where, Ca^2+^ and Mg^2+^ concentrations are expressed in terms of (meq l^−1^).

### Data of Statistical Analysis

The statistical analyses and water samples chemical analysis were carried out using SAS (version 9.2), MINITAB (version 14) and Rockworks (version 15) and AqQA (version 1.1.1) water-quality software, respectively.

## Results and Discussion

**Fig. 2:**
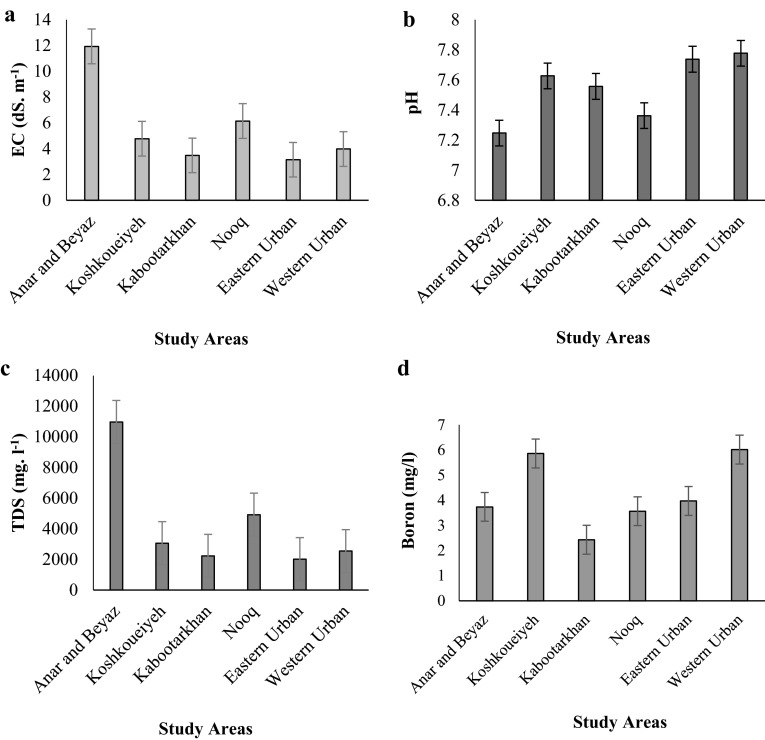
**a** EC (dS/m), **b** pH, **c** TDS (mg/l), and **d** Boron (mg/l) values in study areas

**Fig. 3:**
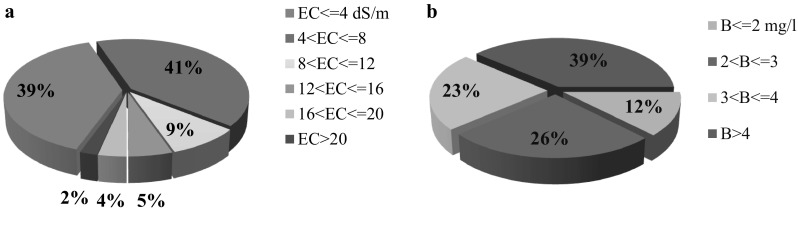
Pie diagram of **a** EC (dS/m), **b** boron concentration of total groundwater samples in study areas

The chemical compositions of the well water samples were analyzed, and the results are presented in average values (Table 1). The results showed that the maximum and the minimum values of EC were observed in Anar-Beyaz and Eastern Urban, respectively (Fig. 2a). According to the degree of restriction on the use for EC, based on FAO guidelines, it is severe (EC > 3 dS m^−1^) in all of areas for agricultural practices (Ayers and Westcot 1985). The maximum permissible value of EC for drinking water is 1.4 dS m^−1^ (WHO 2011). In the study areas, the EC values of water samples were in the range of 3.15–11.9 dS m^−1^, indicating EC values which are higher than the prescribed limit for drinking water. When the experimental values were compared with the standard values recommended by WHO (2011) for drinking water and public health, in all of parts of Rafsanjan area, EC values were found to exceed the prescribed limit of WHO (2011). Figure 3a shows that in 39 % of total water samples, EC values were lower than 4 dS m^−1^. According to the prescribed EC values and also FAO guidelines for irrigation water (1985), in fact, the result showed that the EC values of drinking and irrigation water condition are critical and of serious concern in the study areas.

**Table 1:** Summary statistics of chemical compositions of major ions (mg l^−1^) in the groundwaters of Rafsanjan areas

Variable		EC (dS/m)	pH	Ca	Mg	Na	Cl	HCO_3_	SO_4_	Boron	Total hardness	SAR
				mg l^−1^								
Anar and Beyaz (180)*												
SD		6.03	0.40	17.02	19.23	42.58	65.07	3.23	10.23	2.6	8.50	1.74
Max		28.30	8.27	1600	864	4807	10,635	549	3470.40	17.5	7400	81
Min		1.30	6.22	16	19.20	195.50	35.45	30.50	86.40	1.1	160	5.90
Mean		11.93	7.25	577.28	334.29	1661.62	3918.90	132.61	698.45	3.7	2836.06	14.77
Koshkoueiyeh (171)												
SD		2.64	0.28	4.45	4.05	21.29	24.95	1.32	6.05	3	4.63	4.04
Max		14.89	8.84	440	240	3105	4785.75	381.25	1632	14.5	1850	36
Min		1.03	6.96	16.00	9.60	161.00	63.81	30.50	14.40	1.2	80	4.90
Mean		4.78	7.63	125.40	70.06	799.07	1274.28	90.80	409.21	5.9	605.44	14.02
Kabootarkhan (50)												
SD		1.15	0.18	1.81	11.48	8.37	6.06	1.37	10.10	1.1	1.86	5.96
Max		8.20	8.25	240.00	964.80	1380.00	1382.55	244.00	2918.40	5.6	4200.00	15.40
Min		1.72	7.18	48.00	33.60	266.80	301.33	45.75	124.80	1.1	280.00	6.50
Mean		3.49	7.56	98.08	129.41	509.27	708.86	94.73	612.58	2.4	784.40	8.53
Nooq (361)												
SD		3.71	0.29	6.21	11.89	27.05	36.09	2.77	10.04	2.4	4.00	8.61
Max		32.94	8.63	920.00	1128.00	5878.80	12,407.50	427.00	3782.40	32.3	6800.00	34.00
Min		0.03	6.53	56.00	19.20	345.00	74.45	30.50	67.20	1.7	260.00	6.20
Mean		6.15	7.36	213.16	210.76	882.19	1652.37	129.66	754.78	3.6	1411.09	10.02
Eastern Urban (158)												
SD		1.31	0.26	3.06	4.46	7.92	11.71	1.51	4.88	1	2.15	3.57
Max		6.65	8.69	360.00	276.00	1472.00	2215.63	244.00	2088.00	6.7	2000.00	18.50
Min		0.64	7.16	32.00	19.20	207.00	88.63	30.50	60.00	1.4	200.00	5.60
Mean		3.15	7.74	118.25	90.64	535.22	924.31	85.68	376.63	4	673.28	9.28
Western Urban (120)												
SD		2.02	0.31	4.60	7.50	12.31	19.32	1.47	5.60	3	2.78	5.82
Max		12.33	9.11	580.00	468.00	1897.50	4147.65	244.00	1464.00	23.7	3400.00	17.80
Min		0.92	7.24	16.00	14.40	92.00	141.80	30.50	72.00	1.2	100.00	4.70
Mean		3.98	7.78	130.25	92.83	588.74	1060.83	83.13	386.82	6	712.42	10.30

*SD* standard deviation, *max* maximum, *min* minimum

* Area name (number of sample)

The application of irrigation water with high EC values into the soil introduces salts into the root zone. Plant roots take in water but absorb very little salt from the soil solution (Hussain et al. 2008). Similarly, water evaporates from the soil surface but salts remain behind (Karlberg and de Vries 2004). Liu et al. (2012) reported that both processes result in the gradual accumulation of salts in the root zone, even with low salinity water. This situation may affect the plants in two ways: (1) by creating salinity hazards and water deficiency; and (2) by causing toxicity and other problems (Bustan et al. 2004).

The pH of the water is also an indicator of its quality, and normally ranges from 6.5 to 8.4 (Ayers and Westcot 1985). Based on FAO guidelines in all the areas, pH value is at the optimal level (Fig. 2b). According to the results, pH value in Western Urban was higher than the other areas, and its value for Anar-Beyaz area was lower than that of the other areas. The pH values of groundwater in the study areas varied between 7.24 and 7.77, indicating slightly basic water. These pH values were all in the desirable ranges. According to WHO guidelines, pH <6.5 or >9.2 would markedly impair the potability of drinking water (WHO 2011). The pH usually has no direct impact on human health; however, higher value of pH can increase the scale formation in water pipes and also reduce disinfection potential of chloride (Jamshidzadeh and Mirbagheri 2011). More alkaline water requires a longer contact time or a higher free residual chlorine level at the end of the contact time for adequate disinfection (WHO 2011). For example, at pH 6–8, the free residual chlorine must be 0.4–0.5 mg l^−1^; at pH 8–9, it rises to 0.6 mg l^−1^; and chlorination may become ineffective above pH 9 (WHO 2011).

The results showed that the maximum and minimum values of TDS corresponded to Anar-Beyaz area and Eastern Urban area, respectively (Fig. 2c). Classification of well water based on TDS (Freeze and Cherry 1979; Arumugam and Elangovan 2009) indicates that the primary of the samples are brackish water with TDS values ranging from 2015 to 10,971. The maximum permissible concentration of TDS for drinking water is 1000 mg l^−1^, based on taste considerations (WHO 2011). In this study area, the TDS values of the samples were in the range of 2015–10,972. The large variation in TDS values may be attributed to the variation in geological formations, hydrological processes, and the prevailing mining conditions in the region (Liu et al. 2012). In all the areas, TDS is not permissible for drinking (WHO 2011), and according to the FAO guidelines, it is not good even for irrigation (Ayers and Westcot 1985).

The boron concentrations in the water samples varied between 1.1 and 17.5 mg l^−1^. The boron concentrations in Western Urban and Koshkoueiyeh were more than those in the other areas, respectively, as shown in Fig. 2d. The result showed that boron concentrations in 12 and 88 % of the total water samples were lower than and more than 2 mg l^−1^, respectively (Fig. 3b). Based on FAO guidelines for irrigation water, boron values in groundwater samples have reached toxic level and may cause severe problems to irrigation practices (Ayers and Westcot 1985). The maximum permissible concentration of boron for drinking water is 2.4 mg l^−1^ (WHO 2011). According to the Salehi and Hosseinifard’s (2012) study, the status of boron concentration was toxic in groundwater in Rafsanjan area. Regarding pistachio which is the main production in this area, toxicity of boron caused decline in the pistachio yield in this region. Also the results of Mortazavi et al. (2014) supported these results.

Boron is also present in irrigation waters as unionized boric acid expressed as boron element (B) in mg l^−1^. Boron is an essential element to the plants but a boron content exceeding 1–2 mg l^−1^ in irrigation water may be harmful to most plants and cause severe problems to crops (Ayers and Westcot 1985). However, where present in excessive amounts, it is extremely toxic, but toxicity occurs even at relatively very low concentrations of 0.6 mg l^−1^ (Ayers and Westcot 1985). Toxicity occurs with the uptake of boron from the soil solution. The boron tends to accumulate in the leaves until it becomes toxic to the leaf tissue and results in the death of the plant. In arid regions, boron is considered the most harmful element in irrigation water (Ayers and Westcot 1985).

A guideline value of 200 mg l^−1^ for sodium (Na^+^) was established by WHO (2011) based on taste considerations and concentrations in excess of 200 mg l^−1^ may give rise to unacceptable taste. Also according to the FAO guidelines (Ayers and Westcot 1985), the value of 0–40 meq l^−1^ was established for irrigation. The sodium values of water samples were in the acceptable range in the study areas. The results showed that sodium concentration is important when evaluating the suitability of groundwater for irrigation. High concentrations of Na^+^ are undesirable in water due to adsorption of Na^+^ onto the soil cation exchange sites, dispersion of soil aggregates, and reduction of soil permeability (Gholami and Srikantaswamy 2009). The SAR, which indicates the effect of relative cation concentration on Na^+^ accumulation in the soil, was used for evaluating the sodicity of irrigation water (Liu et al. 2012).

The high concentrations of chloride can give a salty taste to drinking water (WHO 2011). It can increase the rate of corrosion in water pipes (WHO 2011). According to WHO guidelines, the taste thresholds for chloride are in the range of 200–300 mg l^−1^. On average, concentrations in excess of 250 mg l^−1^ can be detected by taste (WHO 2011). The chloride concentration greater than 600 mg l^−1^ would markedly impair the potability of water. This value is the maximum permissible concentration for drinking water. In study areas, the chloride values of groundwater were in the range of 80–7400, indicating salty water. About 95 % of water samples had higher chloride values than the prescribed limit for drinking water. Also according to the FAO (Ayers and Westcot 1985) guidelines, the qualification of chloride is very severe making it unsuitable for drinking in the entire study areas.

Chloride had high solubility in soil and remains in the soil solution, while sulphate and bicarbonate combine with calcium and magnesium, where present, to form calcium-sulphate and calcium-carbonate, which are sparingly soluble compounds. Many fruit trees and other cultivations are susceptible to injury from salt toxicity (Karaivazoglou et al. 2005). Chloride, sodium, and boron are absorbed by the roots and transported to the leaves where they accumulate. In harmful amounts, they result in leaf burn and leaf necrosis (Salehi and Hosseinifard 2012). Moreover, direct contact during sprinkling of water drops with a high chloride content may cause leaf burn in high evaporation conditions (Ayers and Westcot 1985). To some extent, bicarbonate is also toxic. Other symptoms of toxicity include premature leaf drop, reduced growth, and reduced yield. In most cases, plants do not show clear toxicity problems until it is too late to remedy the situation (Hosseinifard et al. 2008). Chloride and sodium ions are both present in the solution. Thus, it is difficult to determine whether the damage caused is due to the one or to the other (Ayers and Westcot 1985).

**Fig. 4:**
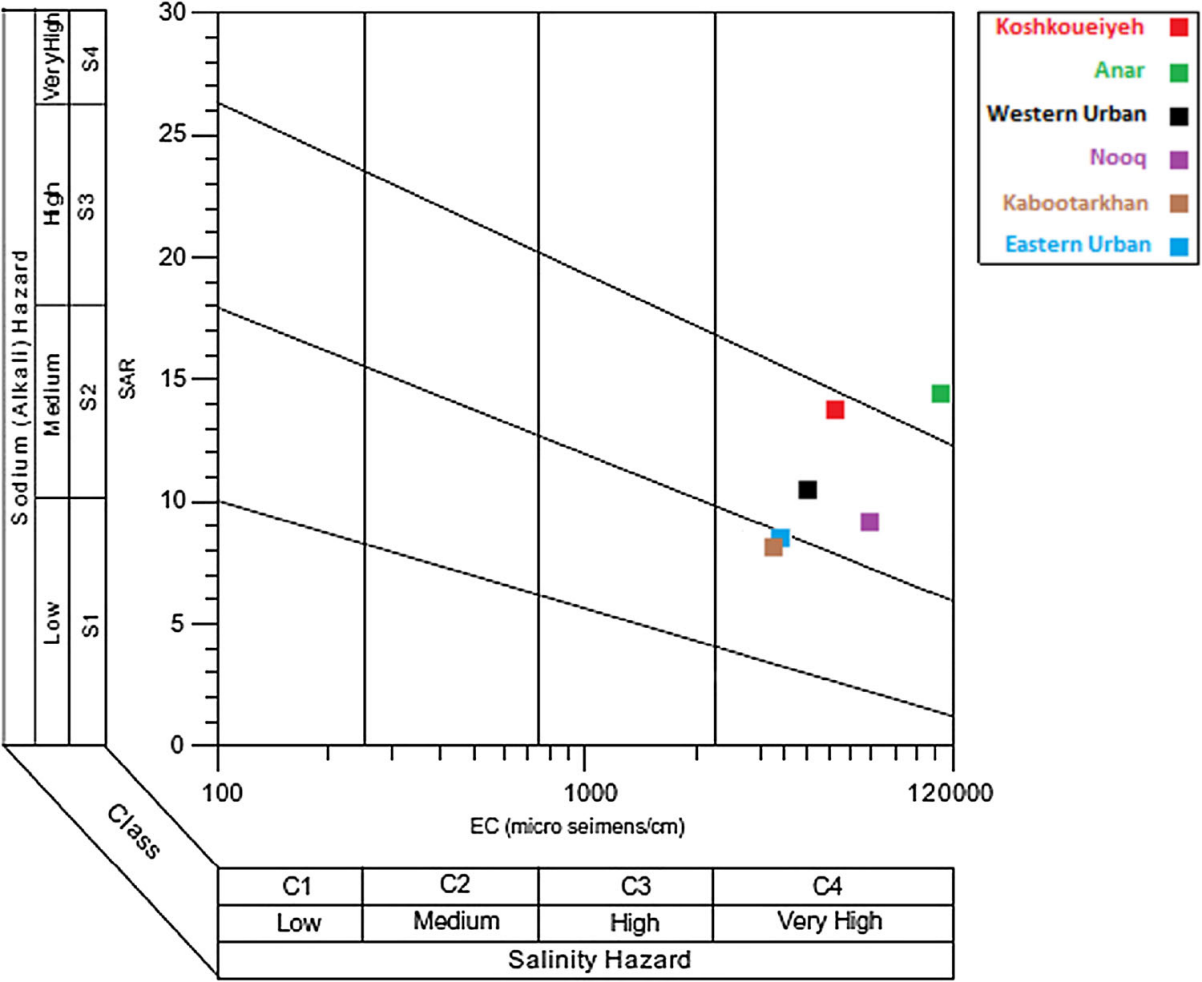
The US salinity hazard diagram (after Richards 1954) for classification of groundwater samples in the study areas

The effect of sodium toxicity is not very clear. However, it has been found that it may cause some direct or indirect damage to many plants (Ayers and Westcot 1985). The usage of water with a high SAR value and low-to-moderate salinity may be hazardous and reduce the soil infiltration rate. The SAR of irrigation water indicates the approximate ESP of a soil with water. In order to identify the availability of waters for irrigation use, the US salinity hazard diagram (after Richards 1954) has been used. This graph is based on the EC and SAR. According to this graph, water salinity classes in all the areas are grouped in C4 class (very high salinity) but water alkalinity classes belong to different classes of SAR (Fig. 4).

In Anar-Beyaz area (C4–S4), water is generally unsatisfactory for irrigation purpose except at low and perhaps medium salinity, where the solution of calcium from the soils or use of gypsum or other amendments may make these waters suitable for use. In Koshkoueiyeh, Nooq, and Western Urban areas (C4–S3), water cannot be used for irrigation without special practices for controlling salinity and alkalinity, such as improvement of drainage, high leaching, and organic matter additions.

In Kabootarkhan and Eastern areas (C4–S2), water is not suitable for irrigation water under ordinary conditions due to very high salinity hazard, and this water will present an appreciable sodium hazard in fine-textured soils having high CEC, especially under low leaching conditions, unless gypsum is present in the soil. Also this water may be used on coarse-textured or organic soils with good permeability.

**Fig. 5:**
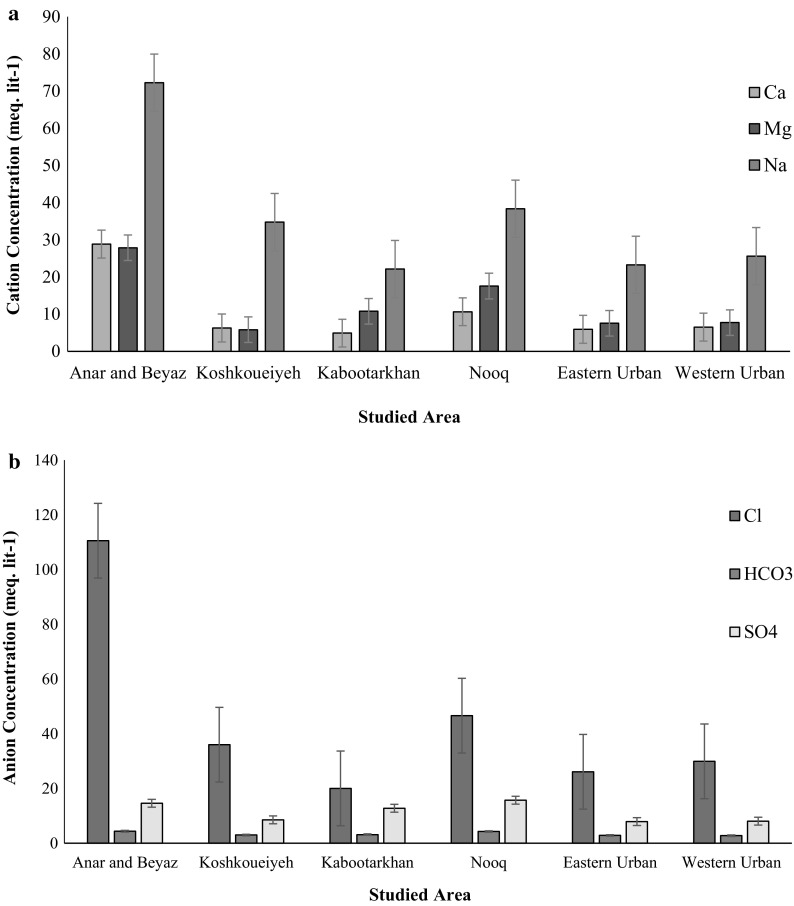
**a** Major cations **b** major anions in the groundwater in study areas

From (Fig. 5a), it was observed that in Anar-Beyaz and Koshkoueiyeh areas, the major cation concentrations in the groundwater are in the decreasing order as Na^+^ > Ca^2+^ > Mg^2+^, and in Kabootarkhan, Nooq, Eastern Urban, and Western Urban areas, the major cations in the groundwater are in the decreasing order as Na^+^ > Mg^2+^ > Ca^2+^. The anions are also arranged in decreasing order as Cl^−^ > ${\text{SO}}_{4}^{2 - }$ > ${\text{HCO}}_{3}^{ - }$ (Fig. 5b). The carbonate concentration in all of areas was very low. Thus, it can be found that, in all the areas, Na cation and Cl anion were dominant in groundwater samples (Fig. 5a, b). These results were similar to the results reported by Salehi and Hosseinifard (2012) that according to the chemical analysis of the groundwater in Rafsanjan areas, mean concentration of the cations is of the order Na^+^ > Ca^2+^ > Mg^2+^, while that for anions is Cl^−^ > ${\text{SO}}_{4}^{2 - }$ > ${\text{HCO}}_{3}^{ - }$.

According to the Table 2, there are statistically significant ($\alpha$ = 0.01) positive correlations between Cl and Na (*r* = 0.993), Cl and Ca (*r* = 0.994), and Cl and Mg (*r* = 0.911). Table 2 reveals that TDS had good significant correlation with all cations and Cl and HCO_3_ anions at the 0.01 level. Also TDS had statistically positive correlation with EC (*r* = 0.998) and had negative correlation with pH (*r* = −0.854) at the 0.01 and 0.05 levels,, respectively. Based on Table 2, correlation coefficients between total hardness (TH) and Ca, Mg, Na, Cl, and HCO_3_ were statistically significant at the 0.01 level (*r* = 0.985, 0.982, 0.954, 0.970, and 0.862, respectively). Boron did not have any correlation with the presented hydrochemical characteristics of groundwater samples (Table 2). The results of many other studies also corroborated the results of the present study (Hosseinifard et al. 2008; Rahnama and Zamzam 2013; Mortazavi et al. 2014).

**Table 2:** Pearson correlation between hydrochemical characteristics of groundwater samples

	Ca	Mg	Na	Cl	HCO_3_	SO_4_	Boron	EC	TDS	pH	TH
Ca	1										
Mg	0.934**	1									
Na	0.977**	0.896*	1								
Cl	0.994**	0.911*	0.993**	1							
HCO_3_	0.782	0.919**	0.802	0.783	1						
SO_4_	0.571	0.813*	0.567	0.547	0.912*	1					
Boron	−0.185	−0.441	−0.090	−0.108	−0.436	−0.670	1				
EC	0.986**	0.930**	0.995**	0.994**	0.827*	0.618	−0.142	1			
TDS	0.993**	0.947**	0.989**	0.994**	0.835*	0.633	−0.189	0.998**	1		
pH	−0.801	−0.921**	−0.827*	−0.799	−0.970**	−0.919**	0.506	−0.848*	−0.854*	1	
TH	0.985**	0.982**	0.954**	0.970**	0.862*	0.698	−0.312	0.976**	0.987**	0.873*	1

** Correlation is significant at the 0.01 level (2-tailed)

* Correlation is significant at the 0.05 level (2-tailed)

WHO guidelines suggested that concentrations of sulfate greater than 400 mg l^−1^ would markedly impair the potability of water; consequently, the maximum permissible value of sulfate in drinking water is 400 mg l^−1^. The sulfate values of samples were in the range of 264.48–1899.4. About 86 % of samples showed higher sulfate values compared with the standard values prescribed by WHO (2011). The presence of sulfate in drinking water may cause bitter taste at concentrations above 250 mg l^−1^ and may contribute to the corrosion of water pipes and distribution systems In natural water; magnesium (Mg) in equilibrium state will adversely affect crop yield (Nagaraju et al. 2006). The magnesium hazard (MH) of irrigation water has been proposed by Szabolcs and darab (1964) and redefined by Raghunath (1987). The MH values exceeding 50 is considered harmful and unsuitable for irrigation use. In the analyzed groundwater samples, the MH values are found to range between 47.9 and 68.8 (Table 3). The average is nearly 50, indicating that groundwater is marginally used for irrigation with little harm associated to Mg in the groundwater.

**Table 3:** Mg hazard values of groundwater samples in study areas

Areas	Anar-Beyaz	Koshkoueiyeh	Kabootarkhan	Nooq	Eastern Urban	Western Urban
Mg hazard	49.1	47.9	68.8	62.2	56.3	54.2

3 $$ {\text{MH}} = \frac{\text{Mg}}{{\left( {{\text{Ca}} + {\text{Mg}}} \right)}} \times 100 $$

The results showed that Mg hazard (MH) status in Kabootarkhan and Nooq water samples had reached critical state, and in the other areas, the MH values were in excess of 50 except in Anar-Beyaz and Koshkoueiyeh areas, as in these areas, groundwater is marginally used for irrigation with little harm associated to Mg.

Hydrochemical properties of groundwater depend on lithology, regional flow pattern of water, and resident time (Domenico 1972). All waters from the viewpoint of chemical compound are divided into three main categories: bicarbonate, sulfate, and chloride types (Chebotarev 1955). The Piper diagram (1944) can be used to identify the type of water. Piper diagrams consist of three parts: two trilinear diagrams along the bottom, and one diamond-shaped diagram in the middle. The trilinear diagrams illustrate the relative concentrations of cations (left diagram) and anions (right diagram) in each sample.

**Fig. 6:**
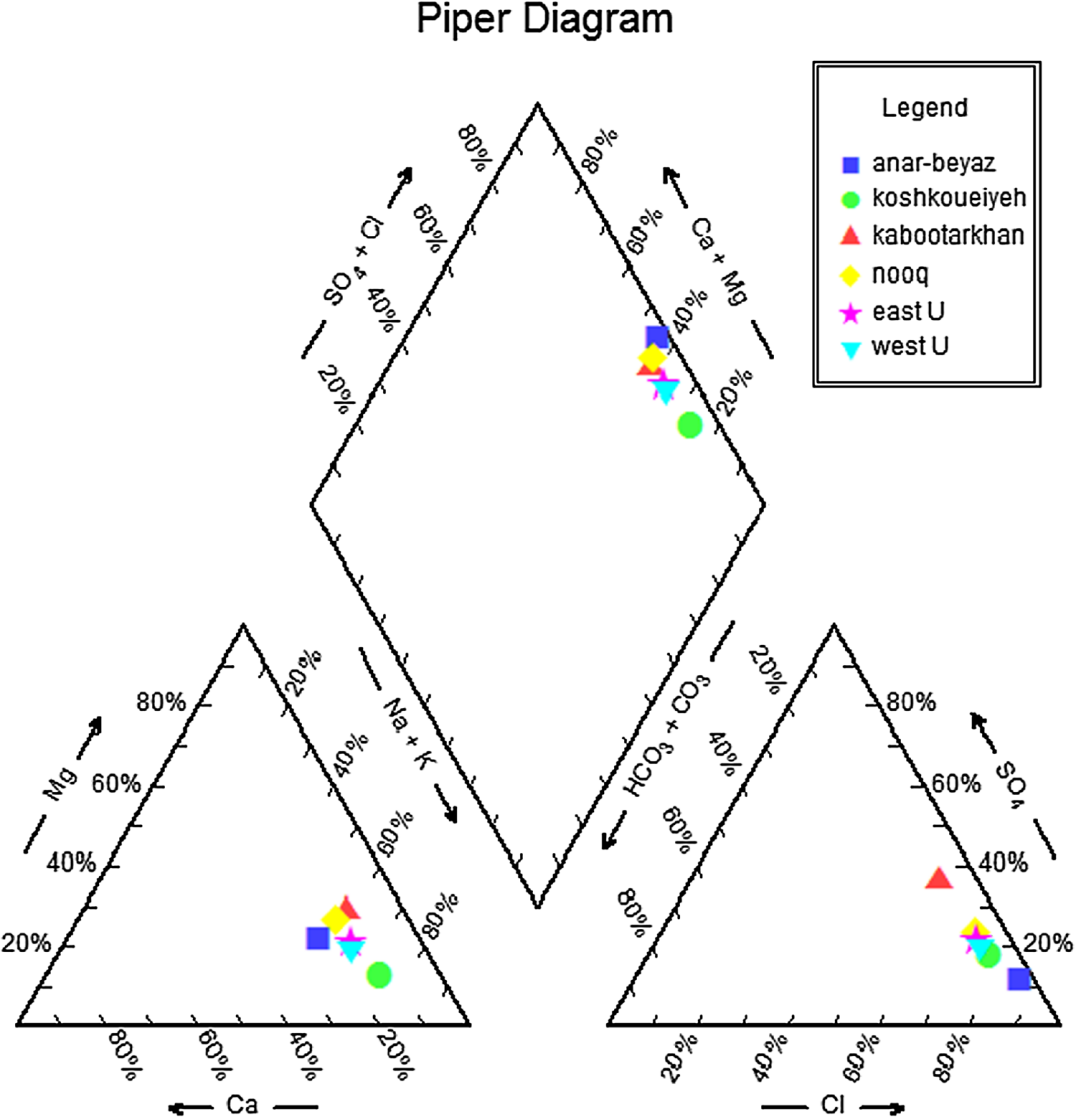
Piper diagram for groundwater samples in the study areas

**Fig. 7:**
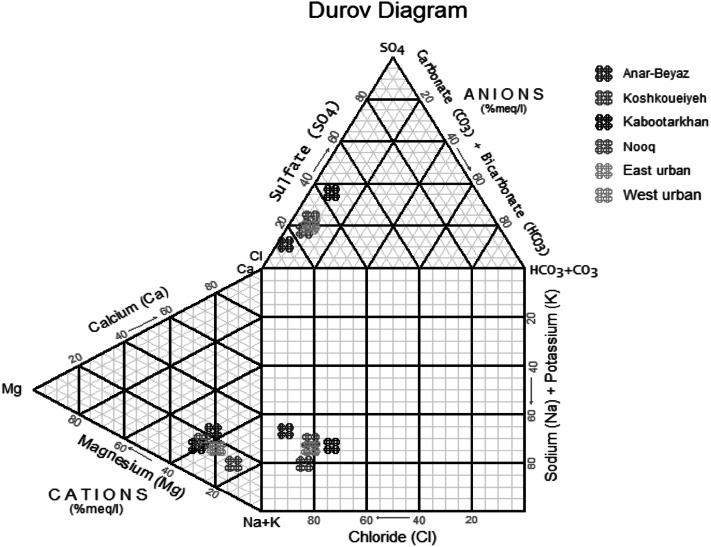
Durov diagram for groundwater samples in the study areas

For the purposes of a Piper diagram, the cations are grouped into three major divisions: sodium (Na^+^) plus potassium (K^+^), calcium (Ca^2+^), and magnesium (Mg^2+^). The anions are similarly grouped into three major categories: bicarbonate (${\text{HCO}}_{3}^{ - }$) plus carbonate (${\text{CO}}_{3}^{2 - }$), sulfate (${\text{SO}}_{4}^{2 - }$), and chloride (Cl^−^). Each sample will be represented by a point in each trilinear diagram; the type of water samples will qualify according to the symbol location in Piper diagram. The Durov Diagram (1948) is an alternative to the Piper diagram. In the two triangles, it plots the major ions as percentages of milliequivalent. The totals of both the cations and anions are set to 100 %, and the data points in the two triangles are projected onto a square grid which lies perpendicular to the third axis in each triangle. According to the Piper and Durov diagrams, it can be found that sodium chloride (Na-Cl) is the main water type in the study areas (Figs. 6 and 7).

Groundwater, which contains numerous natural ions and may be polluted by human activities, seriously influences agricultural utilization. Moreover, irrigation water with excessive ions also impacts the environment. For example, the most common ions found in groundwater are chloride (Cl^−^) and sodium (Na^+^), particularly in coastal aquifers. When water with high Cl^−^ and Na^+^ concentrations is used for irrigation, many plants suffer from toxicity and retardation in growth, resulting in yield reduction (Karaivazoglou et al. 2005; Hosseinifard et al. 2005; Grieve et al. 2006).

**Fig. 8:**
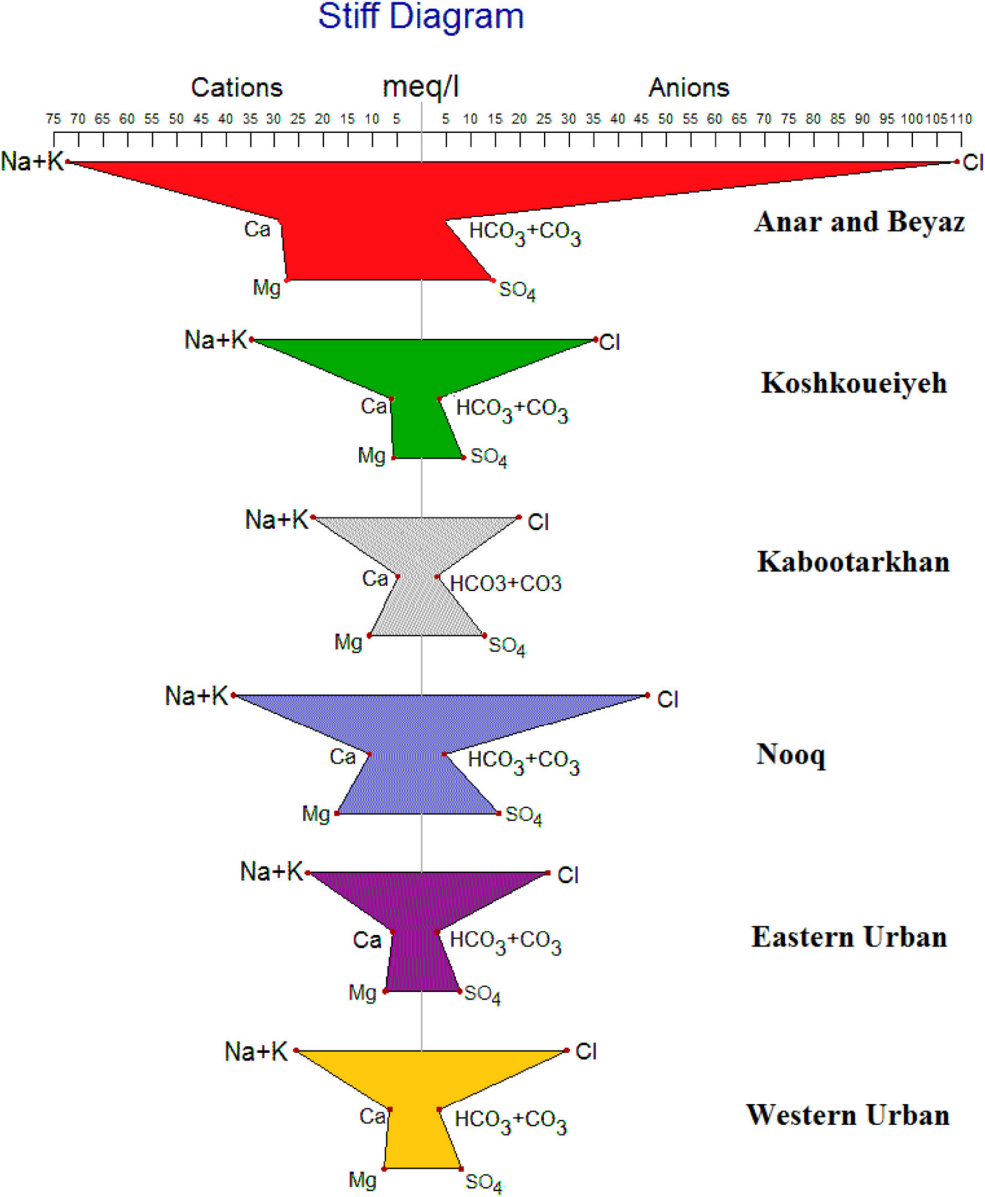
Stiff diagram of Rafsanjan study areas

Stiff diagram shows the composition of a single sample, in terms of common cations and anions, with concentration represented in electrical equivalents. In fact, this diagram investigates dominant cation and anion. Figure 8 shows the (Na + K) cation is the highest among the cations, while the chloride (Cl) ion is the predominant anion in all the areas. This means that the most predominant water type in study areas is the Na-Cl water type. Salehi and Hosseinifard (2012) reported that according to the chemical analysis of the groundwater in Rafsanjan areas, Na^+^ cation and Cl^−^ anion were the dominant ions in groundwater samples.

**Fig. 9:**
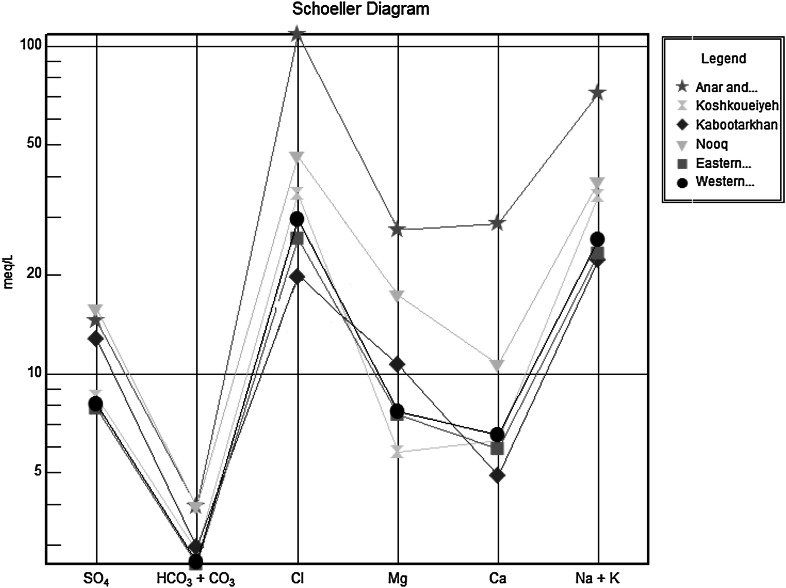
Schoeller diagram of study areas

The Schoeller (1965) diagram is used to study the comparative changes in the concentrations and ratios of water-quality parameters for different samples. The different water-quality parameters are plotted along with their concentrations (meq l^−^) as shown in. It is obvious from Fig. 9 that Anar and Beyaz have the highest concentrations of the major ions and the highest salinity (EC) in the area. Kabootarkhan and Eastern Urban have the lowest concentrations of the major ions, and these areas are therefore classified under freshwater groundwater types.

## Conclusion

Groundwater qualities of different areas in Rafsanjan were studied and assessed. Accordingly, the groundwater samples were collected from wells located at different areas of Rafsanjan region. The physical and chemical parameters of the samples were evaluated to investigate the groundwater quality for irrigation purpose. The results showed maximum and minimum of EC and TDS values corresponded to Anar-Beyaz area and Eastern Urban, respectively. According to the results, pH value in Western Urban was higher than that of the other areas, and its value for Anar-Beyaz area was lower than the other areas; thus, its value is at optimal level in all the study areas. The results showed that 41 and 61 % of total water samples had EC values lower than and more than 4 dS m^−1^, respectively. According to EC values and also FAO guideline for irrigation water, the results showed irrigation water EC statuses in the study areas were indeed critical and serious.

The results showed that Mg hazard (MH) status was critical in the study areas except in Koshkoueiyeh and Anar-Beyaz areas, as in these areas, groundwater is used marginally for irrigation with little harm associated to Mg. Also according to the results, boron concentrations in 12 and 88 % of total water samples were lower than and more than 2 mg l^−1^, respectively. The US salinity hazard diagram shows that salinity hazards in all the areas are regarded to be very high (C4 class), and sodium hazard status is regarded as medium (S2 class) to very high (S4 class). According to the hydrochemistry diagrams, the major cation and anion in groundwater were Na and Cl, respectively, which demands the special management for controlling salinity and sodium hazard for all the studied areas.

In spite of critical status of water quality in the studied areas, farmers are forced to use this water for agriculture practices. Hence, one way of production of crops in these areas is the implant of plants highly tolerant to the salinity and sodicity in this region, such as pistachio tree. Also groundwater quality in these areas was not appropriate for drinking usage and could be harmful to health. The uniform management of groundwater resource for irrigation and drinking uses by the government can be one of the appropriate ways to solve water-quality issues not only in Rafsanjan area, but also in other arid and semi-arid areas.
